# Ditching the Driving: A Cross-Sectional Study on the Determinants of Remote Work From Home for Sign Language Interpreters

**DOI:** 10.3389/frhs.2022.882615

**Published:** 2022-06-17

**Authors:** Gretchen Roman, Vincent Samar, Deborah Ossip, Michael McKee, Steven Barnett, Reza Yousefi-Nooraie

**Affiliations:** ^1^Clinical and Translational Science Institute, University of Rochester Medical Center, Rochester, NY, United States; ^2^Department of Public Health Sciences, University of Rochester Medical Center, Rochester, NY, United States; ^3^Department of Liberal Studies, National Technical Institute for the Deaf, Rochester Institute of Technology, Rochester, NY, United States; ^4^Department of Family Medicine, University of Michigan, Ann Arbor, MI, United States; ^5^Department of Family Medicine, University of Rochester Medical Center, Rochester, NY, United States

**Keywords:** remote sign language interpreting, implementation behavior change, determinants of implementation behavior, COVID-19 pandemic, behavior constructs

## Abstract

**Background:**

The coronavirus disease 2019 (COVID-19) pandemic dramatically impacted the working conditions for sign language interpreters, shifting the provision of interpreting services from onsite to remote. The goal of this cross-sectional study was to examine the perceptions of determinants of remote interpreting implementation from home by sign language interpreters during the pandemic. We hypothesized that interpreters working across the primary settings of staff (agency, government, business, or hospital employees), educational (K-12 or postsecondary), community/freelance (independent contractor), video remote (the two-way connection between onsite participants and remote interpreter), and video relay (three-way telecommunication) would present with differing experiences of the implementation process.

**Methods:**

The Determinants of Implementation Behavior Questionnaire was adapted for sign language interpreters (DIBQ-SLI) and administered to certified interpreters working remotely at least 10 h per week. The DIBQ-SLI included eight constructs (knowledge, skills, self-efficacy, perceived behavioral control, innovation characteristics, organizational resources and support, innovation strategies, and social support) and 30 items. Parametric statistics assessed differences in interpreters' perceptions across settings. Principal component analysis was conducted for data reduction and affirmation of the most critical constructs and items.

**Results:**

One hundred and six interpreters (37 video relay, 27 video remote, 18 educational, 11 community/freelance, 11 staff interpreters, and two from “other” settings) completed the DIBQ-SLI. The video relay and staff interpreters consistently demonstrated the most favorable and the educational interpreters demonstrated the least favorable perceptions. Of the total variance, 58.8% of interpreters' perceptions was explained by organizational (41%), individual (10.7%), and social (7.1%) dimensions. There were significant differences across settings for the organizational and individual principal components; however, no differences were detected for the social principal component.

**Conclusions:**

An administrative infrastructure devoted to ensuring that interpreters receive sufficient managerial support, training, materials and resources, experience with remote interpreting before having to commit, and insights based on the results of their remote work (organizational principal component) may be necessary for improving perceptions. Remote interpreting is expected to continue after the pandemic ends; thus, settings with the least favorable ratings across behavior constructs may borrow strategies from settings with the most favorable ratings to help promote perceptions of the contextual determinants of future remote interpreting implementation.

## Introduction

The United States Bureau of Labor and Statistics, American Time Use Survey reported that employed persons working from home doubled from 2019 to 2020, increasing from 22 to 42% (1). De Meulder et al. (2) conducted three online “living” surveys from April to July 2020 about sign language interpreting in times of coronavirus disease 2019 (COVID-19), with each iteration of the surveys incorporating respondent feedback. In the first survey, 27% of respondents reported working remotely only occasionally prior to the pandemic, while 60% reported never working remotely. The second survey demonstrated a dramatic shift from onsite to remote interpreting with 0% of participants reporting remote work in the last 6 months of 2019 to 100% of participants in April 2020. The respondents predicted the continuation of remote work in the future, with the majority indicating that 25% of their workload would remain remote. Across all three surveys, 63% of the respondents reported never receiving any training on remote interpreting (2). Other recent studies revealed that 64% of interpreters had never worked remotely prior to the pandemic and that 46% did not feel comfortable or prepared for the transition to remote work. Regarding available resources for remote interpreting, only 18% indicated that workshops and/or training were provided by their employer (3). The shift in the overall workforce from onsite to remote due to the pandemic and lack of experience and training of sign language interpreters working remotely beckons the need for examination of interpreters' perceptions of the determinants of implementation behavior upon shifting from onsite to remote work. Because the demand for remote interpreting is expected to continue, such research will be beneficial in facilitating the transition of future interpreters.

In an effort to more effectively implement innovations and evoke change, several studies have recognized the importance of understanding how to define and measure behavior change (4–7). The Theoretical Domains Framework (TDF) was created by an interdisciplinary group of psychological theory, health services research, and health psychology experts (4). The original TDF conceptualized behavior into 12 domains with 100 constructs (4). Cane et al. (5) updated the domain structure by refining and expanding the original 12 behavior domains to 14 with 84 constructs (5). Huijg et al. (6) developed a TDF questionnaire to measure behavior change based on the original 12-domain version (4) and assessed its psychometric properties (8). The Determinants of Implementation Behavior Questionnaire (DIBQ) was used to measure healthcare professionals' perceptions of factors influencing implementation to effectively integrate innovations into routine healthcare practice (specifically physical therapists in Sweden and Denmark delivering various physical activity interventions to patients with arthritis, chronic obstructive pulmonary disease, diabetes, or obesity) (8). Some items were modified from previously published questionnaires (9, 10), while other newly developed items were informed by the literature (4) and results of past studies (6, 11). The DIBQ was expanded from 12 to 18 domains with 26 constructs and reduced from a total of 100 to 93 items (8).

The DIBQ (8) has been previously adapted to measure expectations of the implementation process for a low back pain program in primary care not yet delivered in practice (12). With the aim of including a realistic number of items and evaluating the implementation process at organizational, individual, social, and contextual levels, content experts in clinical or methodological research with backgrounds in the musculoskeletal system or implementation research were asked to rate the relevancy of the 93 DIBQ items. After undergoing confirmatory factor analysis, a 26-item, 10-domain tailored version unique to implementing a low back pain program (DIBQ-t) was developed. The example by Ris et al. (12) demonstrates that the DIBQ can be successfully adapted to a different context and used to guide the examination of interpreters' perceptions upon transitioning from onsite to remote interpreting in this study.

The goal of this study was to examine sign language interpreters' perceptions and experiences of the determinants of remote interpreting implementation from home during the COVID-19 pandemic. To accomplish this, we adapted the DIBQ into the Determinants of Implementation Behavior Questionnaire for Sign Language Interpreters (DIBQ-SLI) to assess the perceptions of contextual determinants of implementation behavior of sign language interpreters throughout the changing work conditions of the pandemic as defined in the TDF (4, 5, 8). We hypothesized that sign language interpreters across the primary interpreting settings of staff, educational, community/freelance, video remote, and video relay (Table 1) would present with differing experiences of the implementation process.

**Table 1:** Definitions of the different primary interpreting settings represented in this study.

**Interpreting setting**	**Definition**
Staff	A full- or part-time salaried position (13), often at a reduced hourly wage when compared with other settings because of benefit eligibility. Staff interpreters can work in a variety of positions, such as an agency, government, business, or hospital employee.
Educational	Sign language interpreting provided between deaf or hard of hearing students in primary or secondary (K-12) and postsecondary education with teachers, school staff, and other students (14). The Registry of Interpreters for the Deaf recognized the Educational Interpreter Performance Assessment (EIPA) from 2007 to 2016 as the necessary credential available for K-12 interpreters, however states now regulate the required standards (15).
Community/freelance	An independent contractor or an interpreter who is self-employed. Community/freelance interpreters manage their own medical and business liability insurance and retirement planning (16). Terms of service for work in the community are negotiated with privately-owned or not-for-profit interpreter referral agencies (17). Community/freelance interpreting assignments often occur with a two-hour payment minimum, but may be charged by the hour (18).
Video remote	A prescheduled or on demand per minute fee-based service with a 15-min payment minimum (or per hour) where two-way communication occurs between deaf or hard of hearing and hearing participants together in one location and an interpreter in a second separate, remote location (19, 20). Services have traditionally been used when there are few qualified community/freelance interpreters in an area and it is cost-prohibitive to have an interpreter travel from a neighboring city (21). Video remote interpreting may be disseminated by a privately-owned interpreter referral agency and can be provisioned in a variety of settings, including educational, legal, medical, or mental health. Video remote interpreters are sometimes able to receive preparation material (if request is made in advance) and able to participate in ongoing assignments, which allows for schema building (18).
Video relay	Federally funded, government regulated three-way telecommunication between deaf or hard of hearing and hearing participants in two different locations and an interpreter in a third separate, remote location (20–22). Video relay service calls vary from personal to professional, across a wide array of contexts (e.g. religious, medical, legal, educational, governmental, etc.), and range in register from formal to intimate. Calls initiated using a video phone can be made from home or using an application on a mobile device, or from a public video phone found in airports, libraries, hospitals, or dormitories. Video relay service providers may offer a ten-minute break to interpreters for each hour of interpreting (23), however break time in between calls is very little. Seconds before a call is placed, minimal exchange of information about call content may or may not be shared (18).

## Materials and Methods

### Ethics Approval and Consent to Participate

This cross-sectional study was approved by the University of Rochester's Research Subjects Review Board (STUDY00005893).

#### Consent for Publication

The Research Subjects Review Board deemed this study exempt; thus, the participants were not required to provide written informed consent. Instead, an information sheet containing study procedures was used.

### Participants

Recruitment materials were shared with not-for-profit interpreting associations, educational institutions, video relay service providers, and interpreter referral agencies. Adults (aged at least 18 years) working remotely as certified sign language interpreters for at least 10 h per week fulfilled the eligibility criteria for completing the DIBQ-SLI. The DIBQ-SLI was administered as part of a larger collective survey instrument used in an overarching study that included measures of the physical and mental health of sign language interpreters across interpreting settings now working remotely from home because of the pandemic (24). Therefore, a power analysis was based on the subjective perception of stress group differences from Rosiner and Shlesinger (25), which indicated that 100 participants would be sufficient to detect a small to medium effect (0.32). We aimed to recruit 120 participants to bolster the likelihood of achieving significant differences across primary settings (Table 1) in interpreters' perceptions of the determinants of implementation behavior upon shifting from onsite to remote work.

### Data Collection

All the participants were able to access a link *via* the recruitment material to the DIBQ-SLI between March and September 2021 using the Research Electronic Data Capture online survey platform (REDCap, Vanderbilt University, Nashville, TN, United States).

#### Outcome Measures

##### Evolution of the Theoretical Domains Framework and the Determinants of Implementation Behavior Questionnaire

The 12 domains in the original TDF included knowledge, skills, social/professional role and identity, beliefs about capabilities, beliefs about consequences, motivation and goals, memory, attention and decision processes, environmental context and resources, social influences, emotion, behavioral regulation, and nature of the behaviors (4). Ten of the original domains remained in the later-refined TDF, the optimism and reinforcement domains were newly added, and the motivation and goals domain was separated into intentions and goals, while the nature of the behavior's domain was dropped (5). With some changes similar to the later-refined TDF (5), eight of the original domains remained in the DIBQ, an optimism domain was newly established, the motivation and goals domain was separated into intentions and goals, and the environmental context and resources domain was divided into five domains (innovation, sociopolitical context, organization, patient, and innovation strategy), and the emotion domain was separated into positive and negative emotions, while the memory, attention, and decision processes domain was combined with nature of the behaviors (8).

##### Determinants of Implementation Behavior Questionnaire for Sign Language Interpreters

Cognitive interviews were conducted by a staff interpreting administrator who worked as a manager of an interpreting services department in an academic medical center and a community/freelance interpreting administrator who worked as a director of a community interpreter referral service housed in a not-for-profit organization. The impact of the pandemic on the provision of sign language interpreting services and the administrators' observations of interpreters' readiness to change from onsite to remote interpreting were discussed. Based on the information gained from the cognitive interviews and relevant literature (12, 26), investigators discussed the constructs within the domains and relevant items most pertinent to the change in work conditions for sign language interpreters during the pandemic, and the DIBQ (8) was adapted into a 30-item, 7-domain DIBQ-SLI with eight constructs (Table 2). Items from the domains (related constructs) of knowledge (knowledge), skills (skills), beliefs about capabilities (self-efficacy and perceived behavior control), innovation (innovation characteristics), organization (organizational resources and support), innovation strategy (innovation strategies), and social influences (social support) were converted to the context of remote interpreting. For example, an item in the skills constructs on the DIBQ that was conveyed as “I have been trained in delivering [physical activity intervention] following the guidelines” was converted to “I have been trained in delivering sign language interpreting services remotely.” As another example, an item in the organizational resources and support construct that was conveyed as “The management of the organization I work in is helpful with delivering [physical activity intervention] following the guidelines” was converted to “The management of the organization where I work is helpful with delivering sign language interpreting services remotely.” The knowledge and skills domains, respectively, measured the sign language interpreter's awareness of the existence of remote interpreting and their “ability or proficiency acquired through practice (5, 8, 27).” The beliefs about capabilities domain analyzed the “acceptance of the truth, reality, or validity about an ability, talent, or facility that a person can put to constructive use (5, 8, 27).” The innovation, organization, and innovation strategy domains, respectively examined any characteristics of the innovation, organization, and innovation strategy “that discourages or encourages the development of skills or abilities, independence, social competence, and adaptive behavior (5, 8, 27).” The social influences domain measured “those interpersonal processes that can cause individuals to change their thoughts, feelings, or behaviors (5, 8, 27)” The respondents were asked to describe their impressions of working remotely as a sign language interpreter over the past week. Most answers adhered to a seven-point Likert scale ranging from −3 (strongly disagree) to +3 (strongly agree). Exceptions were two items in the perceived behavior control construct that ranged from −3 (very difficult) to +3 (very easy).

**Table 2:** Determinants of Implementation Behavior Questionnaire (7) for sign language interpreters (DIBQ-SLI).

**Describe your feelings and behaviors working remotely as a sign language interpreter over the past week**.								
**DIBQ-SLI domain**	**DIBQ-SLI construct**	**−3** **strongly disagree**	**−2**	**−1**	**0** **neither disagree or agree**	**1**	**2**	**3** **strongly agree**
**Knowledge**	**Knowledge**							
**Item**								
I know how to interpret when working remotely as a sign language interpreter								
**Skills**	**Skills**							
**Items**								
I have been trained in delivering sign language interpreting services remotely								
I am practiced in delivering sign language interpreting services remotely								
I have the skills to deliver sign language interpreting services remotely								
**Beliefs about capabilities**	**Self-efficacy**							
**Items**								
I am confident that I can interpret remotely as well as I can interpret onsite								
I am confident that I can interpret remotely even when other sign								
language interpreters are not								
Even when there is little time, I am confident that I can transition from interpreting								
onsite to interpreting remotely								
I am confident I can interpret remotely even when Deaf and hearing consumers								
are not motivated to receive the interpreting services remotely								
**DIBQ-SLI domain**	**DIBQ-SLI construct**	**−3** **very difficult**	**−2**	**−1**	**0** **neither difficult or easy**	**1**	**2**	**3 very easy**
**Beliefs about capabilities**	**Perceived behavior control**							
**Items**								
I have control over working remotely as a sign language interpreter								
For me, delivering sign language interpreting services remotely is								
For me, reporting about my delivery of sign language interpreting services remotely								
to the organization where I work is								
**DIBQ–SLI domain**	**DIBQ–SLI construct**	**−3** **strongly disagree**	**−2**	**−1**	**0** **neither disagree or agree**	**1**	**2**	**3** **strongly agree**
**Innovation**	**Innovation characteristics**							
**Items**								
It is possible to tailor sign language interpreting services delivered remotely to the								
needs of Deaf and hard of hearing consumers								
It is possible to tailor sign language interpreting services delivered remotely to the								
needs of the interpreter								
It takes little time to deliver sign language interpreting services remotely								
Working remotely as a sign language interpreter is compatible with daily practice								
It is simple to deliver sign language interpreting services remotely								
**Organization**	**Organizational resources and support**							
**Items**								
In the organization where I work, all the necessary resources are available to								
interpret remotely								
I can count on support from the management where I work when interpreting remotely								
gets tough								
The management of the organization where I work is willing to listen to my								
problems with delivering sign language interpreting services remotely								
The management of the organization where I work is helpful with delivering								
sign language interpreting services remotely								
**Innovation strategy**	**Innovation strategies**							
**Items**								
The organization where I work provides training to sign language interpreters								
who deliver services remotely								
The organization where I work provides the possibility to experience delivering								
sign language interpreting services remotely before interpreters need to commit to it								
The organization where I work provides sufficient materials and resources								
about interpreting remotely								
The organization where I work provides assistance to sign language interpreters								
delivering services remotely								
The organization where I work organizes meetings for sign language interpreters								
who work remotely								
The organization where I work provides sufficient financial reimbursement								
to sign language interpreters for delivery of services remotely								
The organization where I work provides insights into the results of my work as								
a sign language interpreter delivering services remotely								
**Social influences**	**Social support**							
**Items**								
I can count on support from other sign language interpreters who work								
remotely when working remotely gets tough								
Sign language interpreters who also work remotely are willing to listen to my								
problems with delivering interpreting services remotely								
Sign language interpreters who also work remotely are helpful with delivering								
interpreting services remotely								

*−3 to +3 indicates negative to positive behaviors; Determinants of Implementation Behavior Questionnaire for sign language interpreters (DIBQ-SLI)*.

### Data Processing and Analysis

Cronbach's alpha was used to measure the internal consistency of non-dichotomous items in each construct and, overall, on the DIBQ-SLI. Differences across interpreting settings for the aggregate determinants of implementation behavior constructs were separately evaluated by analysis of covariance (ANCOVA) while adjusting for the covariates of age, sex, hearing status, race, and education. Principal component analysis (PCA) on the correlation matrix of the DIBQ-SLI items with varimax rotation was conducted as a preliminary tool for confirmation of the most prominent constructs and items relevant to the study context. Differences across interpreting settings for principal component scores were also separately evaluated by ANCOVA while adjusting for the mentioned covariates. If significant differences were detected across settings, *post hoc* analyses were performed by pair-wise comparisons of covariance adjusted means to assess for differences across specific interpreting settings. A false discovery rate correction *via* a custom MATLAB code (MathWorks, Natick, MA) was used to control for type I errors across multiple comparisons (28). Statistical analyses were performed using SPSS (v.27, IBM, Armonk, NY, United States) with a significance of *p* < 0.05.

## Results

### Participants

A total of 106 sign language interpreters (27.8 ± 0.9 remote work hours per week, aged 45.7 ± 1 years; 80.2% women; 99.1% hearing; 86.8% White) completed the DIBQ-SLI. The participants represented the primary interpreting settings of video relay (34.9%, *n* = 37), video remote (25.5%, *n* = 27), educational (17%, *n* = 18), community/freelance (10.4%, *n* = 11), staff (10.4%; *n* = 11), and “other” (1.9%; *n* = 2).

### Determinants of Implementation Behavior Constructs

The internal consistency within each of the eight constructs (Table 3) was good to excellent and, overall, across the 30-item DIBQ-SLI was excellent (Cronbach's alpha 0.734 to 1 and overall 0.947). The covariance-adjusted aggregates for the respective items within each determinant of implementation behavior construct across the interpreting settings were calculated (mean ± SEM; Table 3). Overall, when describing the determinants of remote interpreting implementation, sign language interpreters scored most to least favorable across the constructs of knowledge (2.44 ± 0.12), skill (2 ± 0.12), self-efficacy (1.75 ± 0.13), social support (1.66 ± 0.16), perceived behavioral control (1.15 ± 0.14), organizational resources and support (1.02 ± 0.17), innovation characteristics (0.86 ± 0.13), and innovation strategies (−0.08 ± 0.17). As shown in Table 3, except for social support, there were significant differences [F (4, 94) ≥ 2.68, *p* ≤ 0.036] across interpreting settings for all determinants of implementation constructs. For *post hoc* comparisons, interpreters in the video relay setting (*p* ≤ 0.030) consistently had positive perceptions or higher ratings than in the educational interpreting setting across all the significant aggregate determinants of implementation behavior constructs. Staff interpreters (*p* ≤ 0.03) also had higher innovation characteristics, organizational resources support, and innovation strategies construct ratings than educational interpreters. Interpreters in the video remote setting (*p* ≤ 0.047) also had higher skills and innovation strategies when compared with the educational setting. Video relay interpreters had the most favorable perceptions across skills, self-efficacy, perceived behavioral control, and innovation strategies implementation behavior constructs. Staff interpreters had the most favorable ratings for innovation characteristics and organizational resources and support. Community/freelance interpreters demonstrated the most favorable ratings across knowledge and social support. Educational interpreters consistently demonstrated the least favorable perceptions across all determinants of implementation behavior constructs.

**Table 3:** Covariance-adjusted aggregate determinants of implementation behavior and principal component scores (mean ± SEM) across interpreting settings (*significant *post hoc* comparisons).

**DIBQ-SLI** **domain**	**DIBQ-SLI construct**	**Staff** **(*n* = 11)**	**Educational** **(*n* = 18)**	**Community/freelance** **(*n* = 11)**	**Video remote ** **(*n* = 27)**	**Video relay** **(*n* = 37)**	**F(4, 94)**	***p***	**Cronbach's** **alpha**
**Knowledge**	**Knowledge**	2.44 ± 0.34	1.75 ± 0.26*	2.83 ± 0.34	2.49 ± 0.21	2.71 ± 0.18*	2.680	0.036	1.000
		Video relay had higher knowledge (*p* = 0.030) ratings when compared with educational							
**Skills**	**Skills**	2.17 ± 0.34	1.15 ± 0.26*	2.23 ± 0.34*	2.00 ± 0.21*	2.43 ± 0.18*	4.153	0.004	0.734
		Community/freelance (*p* = 0.047), video remote *(p* = 0.047), and video relay (*p* = 0.001) had higher skills than educational							
**Beliefs about capabilities**	**Self-efficacy**	1.76 ± 0.36	1.12 ± 0.27*	1.96 ± 0.35	1.65 ± 0.22	2.28 ± 0.19*	3.361	0.013	0.825
	**Perceived behavioral** **control**	1.47 ± 0.40	0.35 ± 0.30*	0.82 ± 0.39	1.23 ± 0.24	1.87 ± 0.21*	4.703	0.002	0.780
		Video relay measured greater self-efficacy (*p* = 0.010) and perceived behavioral control (*p* = 0.010) than educational							
**Innovation**	**Innovation characteristics**	1.46 ± 0.37*	0.11 ± 0.28*	0.39 ± 0.37	0.86 ± 0.23	1.45 ± 0.20*	4.753	0.002	0.829
		Staff (*p* = 0.030) and video relay (*p* = 0.010) had higher innovation ratings when compared with educational							
**Organization**	**Organizational resources and support**	1.95 ± 0.49*	−0.12 ± 0.37*	0.81 ± 0.49	0.80 ± 0.30	1.34 ± 0.26*	4.635	0.002	0.919
		Staff (*p* = 0.005) and video relay (*p* = 0.005) had greater organization ratings when compared with educational							
**Innovation strategy**	**Innovation strategies**	0.55 ± 0.47*	−1.30 ± 0.36*	−0.56 ± 0.47*	−0.04 ± 0.29*	0.93 ± 0.25*	7.124	0.001	0.903
		Staff (*p* = 0.015), video remote (*p* = 0.020), and video relay (*p* = 0.001) settings had higher innovation							
		Strategy ratings than educational; video relay also had higher innovation strategy ratings than community/freelance							
		(*p* = 0.020) and video remote (*p* = 0.026)							
**Social influences**	**Social support**	1.73 ± 0.47	1.31 ± 0.35	1.93 ± 0.46	1.49 ± 0.28	1.84 ± 0.25	0.555	0.696	0.902
**Principal component**		**Staff** **(*****n*** **=** **11)**	**Educational** **(*****n*** **=** **18)**	**Community/** **freelance** **(*****n*** **=** **11)**	**Video remote** **(*****n*** **=** **27)**	**Video relay** **(*****n*** **=** **37)**	**F(4, 94)**	***p***	
**Organizational**		0.48 ± 0.29*	−0.66 ± 0.22*	−0.52 ± 0.29*	−0.06 ± 0.18	0.41 ± 0.15*	5.594	0.001	
		Staff and video relay had higher organizational component scores than educational (staff *p* = 0.010, video relay *p* = 0.010)							
		and community/freelance interpreters (staff *p* = 0.038, video relay *p* = 0.017)							
**Individual**		−0.04 ± 0.31	−0.63 ± 0.24*	0.08 ± 0.31	−0.05 ± 0.19	0.34 ± 0.17*	2.909	0.026	
		Video relay (*p* = 0.010) had higher individual component scores than educational							
**Social**		−0.05 ± 0.33	−0.03 ± 0.25	0.55 ± 0.33	−0.12 ± 0.20	−0.05 ± 0.17	0.832	0.508	

*−3 to +3 indicates negative to positive behaviors; Determinants of Implementation Behavior Questionnaire for sign language interpreters (DIBQ–SLI)*.

### Principal Component Analysis

The eigenvalues of the scree plot (λ) tended to flatten out after the third principal component. Therefore, three principal components were retained and rotated to a simple structure, with the first (41%; λ=12.29), second (10.7%; λ=3.22), and third (7.1%; λ=2.13) principal components explaining 58.8% of the total variance. The first component strongly represented the organizational resources and support and the innovation strategies determinants of implementation behavior, the second represented self-efficacy and skills, and the third represented social support (Table 4). We ascertained that these components respectively resembled the organizational, individual, and social dimensions of sign language interpreters' perceptions and experiences upon transitioning from onsite to remote work. Specifically, the organizational resources and support and the innovation strategies items with the highest component loadings indicate that the organizational dimension can be defined as receiving sufficient managerial support, training, materials, and resources, experience with remote interpreting before having to commit, and insights based on the results of interpreters' remote work. The self-efficacy and skills items with the highest component loadings indicate that the individual dimension can be defined as interpreters' beliefs in their abilities to transition from onsite to remote interpreting regardless of what other interpreters were doing, as well as interpreters' beliefs in their interpreting skills and in their abilities to practice delivering sign language interpreting remotely. The social support items with the highest component loadings indicate that the social dimension can be defined as problem-solving with and support received from fellow remote interpreting colleagues.

**Table 4:** Top component loadings for principal components 1, 2, and 3 from the rotated component matrix.

**DIBQ–SLI domain**	**DIBQ–SLI construct**	**Component loadings for principal component 1**	**DIBQ–SLI domain**	**DIBQ-SLI construct**	**Component loadings for principal component 2**	**DIBQ-SLI domain**	**DIBQ-SLI construct**	**Component loadings for principal component 3**
**Organization**	**Organizational resources and support**		**Beliefs about capabilities**	**Self-efficacy**		**Social influence**	**Social** **support**	
**Items**			**Items**			**Items**		
I can count on support from the		0.813	I am confident that I can interpret		0.736	I can count on support from		0.838
management where I work when			remotely as well as I can interpret			other sign language interpreters		
interpreting remotely gets tough			onsite			who work remotely when working		
						remotely gets tough		
The management of the organization		0.717	I am confident that I can interpret		0.825	Sign language interpreters who		0.874
where I work is willing to listen to my			remotely even when other sign			also work remotely are willing to		
problems with delivering sign language			language interpreters are not			listen to my problems with delivering		
interpreting services remotely						interpreting services remotely		
The management of the organization		0.807				Sign language interpreters who also		0.819
where I work is helpful with delivering						work remotely are helpful with		
sign language interpreting						delivering interpreting services		
services remotely						remotely		
			**Skills**	**Skills**				
			**Items**					
			I am practiced in delivering sign		0.707			
			language interpreting services remotely					
**Innovation strategy**	**Innovation strategies**							
**Items**								
The organization where I work		0.796						
provides training to sign language								
interpreters who deliver services								
remotely								
The organization where I work provides		0.795	I have skills to deliver sign language		0.749			
the possibility to experience delivering			interpreting services remotely					
sign language interpreting services								
remotely before interpreters need to								
commit to it								
The organization where I work provides		0.830						
sufficient materials and resources about								
interpreting remotely								
The organization where I work provides		0.857						
assistance to sign language interpreters								
delivering services remotely								
The organization where I work provides		0.776						
insights into the results of my work as a								
sign language interpreter delivering								
services remotely								

*Determinants of Implementation Behavior Questionnaire for sign language interpreters (DIBQ-SLI)*.

The covariance-adjusted organizational, individual, and social principal component scores across the interpreting settings were calculated (mean ± SEM; Table 3). Overall, the organizational, individual, and social component scores were −0.7 ± 1, −0.06 ± 0.11, and.06 ± 0.11, respectively. There were significant differences across interpreting settings for the organizational [F(4, 94) = 5.594, *p* = 0.001] and individual [F(4, 94) = 2.909, *p* = 0.026] dimensions; however, no differences were detected for the social dimension. For the *post hoc* comparisons, video relay interpreters (*p* ≤ 0.01) consistently had positive perceptions or higher ratings than the educational interpreting setting across all the significant principal component scores. The video relay setting (*p* = 0.017) also had higher organizational component scores than the community/freelance setting. Staff interpreters also had higher organizational component scores than the educational (*p* = 0.01) and community/freelance interpreters (*p* = 0.038). The staff interpreters demonstrated the most favorable organizational component scores, the video relay interpreters had the highest individual component scores, and the community/freelance interpreters demonstrated the most favorable social component scores. The video remote interpreters had the lowest social component scores; otherwise, the educational interpreters consistently demonstrated the least favorable scores across organizational and individual components.

## Discussion

The goal of this study was to examine sign language interpreters' perceptions of the determinants of remote interpreting implementation from home during the COVID-19 pandemic. A descriptive analysis of the determinants of implementation behavior was performed, and the findings were compared across interpreting settings. The video relay interpreters had the most favorable ratings for skills, self-efficacy, perceived behavior control, and innovation strategies. The staff interpreters had the most favorable ratings for innovation characteristics and organizational resources and support, while the knowledge and social support constructs were rated most favorably by the community/freelance interpreters. Outside of the fairly equivalent distribution of ratings across settings for social support, the results from this study were in support of our hypothesis that interpreters across settings would present with differing experiences of the implementation process. Interpreters in the video relay setting consistently had more positive perceptions than interpreters in the educational setting across all significant aggregate determinants of implementation behavior constructs. The staff interpreters also had higher innovation characteristics, organizational resources, and support, and innovation strategies construct ratings than the educational interpreters. The video remote interpreters also had higher skills and innovation strategies when compared with the educational setting. Higher ratings may signify that conditions in the interpreting setting were more conducive for the remote interpreting implementation behavior, but not necessarily. Our ability to detect causation is limited by the cross-sectional design; however, this research can now inform future studies with sign language interpreters exploring similar perceptions of behavior change.

To provide a broader understanding of the interrelatedness across the DIBQ-SLI, we conducted PCA for data reduction and affirmation of the most prominent constructs and items. Because multiple items from different DIBQ-SLI constructs loaded on one component, differences across interpreting settings in the component scores reveal differences in the underlying dimension shared by these items. Component loadings in this work were higher than those reported in the DIBQ-t (12), possibly indicating greater interrelatedness of the constructs and items within the dimensions for ensuring positive perceptions of remote interpreting implementation compared to implementation of a low back pain program. We found that items most pertinent to change in work conditions for sign language interpreters during the pandemic clustered on components consistent with what was selected by Ris et al. (12) to measure expectations of the implementation process (12). We expected the components to represent the organizational, individual, and social dimensions. If these dimensions did not appear in the PCA, it would call into question the construct validity of the DIBQ-SLI items for the interpreter population.

The first principal component resembled an organizational dimension, explained the greatest amount of variance in the DIBQ-SLI, and demonstrated a substantial impact on interpreters' perceptions and experiences of the determinants of implementation. The organizational dimension revealed significant differences across interpreting settings; specifically, the staff and video relay settings had more positive perceptions than both the educational and community/freelance settings. Educational interpreters who responded to the survey were not receiving sufficient managerial support, training, materials and resources, experience with remote interpreting before having to commit, financial reimbursement, and insight when compared with staff and video relay interpreters, to believe that it is possible to tailor interpreting services to be delivered remotely. Strategies for improved perceptions of remote interpreting implementation in the educational setting may include ensuring an administrative infrastructure devoted to addressing the concerns of the educational interpreting team. Educational interpreting administrators might consider inquiring with staff interpreting departments and video relay service providers and, as appropriate, emulate a similar administrative infrastructure. Due to the self-employed nature of the community/freelance interpreters, they are unable to reap a similar support infrastructure when compared to the staff and video relay settings. Community/freelance interpreters may be able to dialogue openly with one another and interpreter referral agencies with whom they maintain independent contracts to ensure that organizational-level standards are beneficial for all.

The second principal component resembled an individual dimension within the DIBQ-SLI, which was compared across settings. Interpreters' beliefs in their abilities to transition from onsite to remote regardless of what other interpreters were doing, as well as interpreters' beliefs in their interpreting skills and in their abilities to practice delivering sign language interpreting remotely, were seemingly critical. Interpreters in the video relay setting likely had more favorable conditions across the self-efficacy and skills constructs, probably because of their experience with the virtual work environment in call centers prior to working remotely from home. One option for educational interpreters lacking self-efficacy and with a desire to practice their remote interpreting skills might be to seek mentorship. Past evidence has explored best teaching practices of video remote interpreting in interpreter education programs (18), however, further integration of remote interpreting practices and training into interpreter education, in general, could also help to promote interpreters' self-efficacy and skills when working remotely.

Lastly, the third principal component resembled a social dimension. Although the community/freelance interpreters demonstrated the most favorable experiences of the implementation process for the third principal component, the social dimension was fairly equally distributed, as there were no significant differences found across interpreting settings. To foster perceptions, interpreting administrators and interpreting leaders across settings could intentionally create virtual environments for remote interpreting colleagues to engage with one another and offer peer support. Even though the social support construct was fairly high performing as it ranked fourth out of the eight constructs studied, virtual team-building activities will still likely be needed to assist interpreters in maintaining the social dimension. Another suggestion to help mitigate elements of professional isolation for interpreters who are predominantly remote workers is to intersperse face-to-face or onsite interpreting (29).

The evidence from this study for tool validation of the DIBQ-SLI appears promising, but more studies are needed. Three of the four organizational resources and support items, five of the 11 innovation strategies items, two of the four self-efficacy items, and two of the three skills items had the highest component loadings in the first and second principal components, indicating that the other items from these constructs might not be needed in future iterations of the DIBQ-SLI. Social support included three items, and all three had the highest component loadings in the third principal component. Initial data reduction and affirmation did not include the three items from perceived behavioral control, five items from innovation characteristics, and one item from knowledge, indicating that these constructs may not be needed in future iterations of the DIBQ-SLI. Interpreters already seemed to have developed a sense of control from onsite interpreting (26) that they were able to transfer over to the remote context. Interpreters' abilities to tailor sign language interpreting to varying needs, thoughts on whether remote interpreting was simple to deliver or compatible with daily practice, and knowledge of working remotely all seemed to be less critical for ensuring positive perceptions of the contextual determinants of implementation. Fear and concerns regarding the risk of contracting COVID-19 did not offer interpreters the choice of whether or not remote interpreting was possible or if they had the knowledge to be successful; rather, interpreters likely sought out remote interpreting because of the heightened demand and need for security amid all the uncertainty from the pandemic.

A broad perspective of individual-level determinants of remote interpreting implementation by way of the DIBQ-SLI was presented in this study; however, there were a few limitations. First, readers are cautioned not to construe individual-level ratings as being reflective of organizational-level perceptions and vice versa (30). Second, the positive perceptions of determinants of implementation and component scores gathered in this study do not necessarily reflect that the actual implementation of remote interpreting was better, as actual implementation measures were not gathered. Third, the authors recognize that strategies employed in one context or interpreting setting to address problematic determinants of implementation may not be transferrable to another context or interpreting setting. Fourth, the DIBQ-t (12) had 26 items and ten domains compared to our 30-item, 7-domain version with eight constructs. The items on each measure differed slightly, so comparisons between this study and previous ones (12) should be made cautiously. Fifth, this study combined K-12 and postsecondary interpreters to represent the educational interpreting setting. Postsecondary or university and college settings likely have access to more resources and technical support to assist interpreters in their transition from onsite to remote work. With the least favorable ratings across all determinants of behavior constructs, future research garnering greater insights on the differences in determinants of implementation between K-12 and postsecondary interpreting is needed to inform ongoing and future remote educational interpreting needs. Finally, the sample sizes represented across the primary interpreting settings were small. While readers are cautioned about deriving generalizations using these limited data as the study may be underpowered, the internal consistency within each domain and overall were good to excellent and comparable to the DIBQ (Cronbach's alpha 0.68 to 0.93) (8), and the DIBQ-t (Cronbach's alpha 0.717 to 1 and overall 0.896) (12). Our findings of significance across specific interpreting settings can now be used to power and inform future studies.

## Contributions to the Field

Roughly 60% of sign language interpreters have never worked remotely prior to the COVID-19 pandemic and have never received any training on remote interpreting. This study adapted the DIBQ to assess perceptions and experiences of the determinants of implementation across interpreting settings throughout the changing work conditions of the pandemic. Interpreters scored most to least favorable across the constructs of knowledge, skill, self-efficacy, social support, perceived behavioral control, organizational resources and support, innovation characteristics, and innovation strategies. The video relay interpreters consistently demonstrated more favorable perceptions of the determinants of implementation than the educational interpreters. Organizational resources and support, innovation strategies, self-efficacy, skills, and social support had the highest component loadings across the organizational, individual, and social dimensions, and thus were deemed the most prominent constructs relevant to the context of the study. The staff and video relay interpreters had greater organizational dimensions than the educational and community/freelance settings, and the video relay setting had a greater individual dimensions than the educational interpreters. This study reinforced that the DIBQ can be modified across different contexts to evaluate perceptions of the implementation process. Since the remote interpreting demand is expected to continue, these results can facilitate the transition of future interpreters from onsite to remote work.

## Data Availability Statement

The original contributions presented in the study are included in the article/Supplementary Material, further inquiries can be directed to the corresponding author.

## Ethics Statement

The studies involving human participants were reviewed and approved by Research Subjects Review Board at the University of Rochester. Written informed consent for participation was not required for this study in accordance with the national legislation and the institutional requirements.

## Author Contributions

GR, VS, SB, and RY-N: conceptualization and methodology. GR: data curation, project administration, and writing—original draft. GR and VS: formal analysis. GR, VS, MM, DO, SB, and RY-N: visualization. GR, VS, DO, MM, SB, and RY-N: writing—review and editing. All authors contributed to the article and approved the submitted version.

## Funding

The project described in this publication was supported by the University of Rochester CTSA award number TL1 TR002000 from the National Center for Advancing Translational Sciences of the National Institutes of Health (GR) and the University of Rochester Center for Community Health and Prevention Mini-Grant (GR). The content is solely the responsibility of the authors and does not necessarily represent the official views of the National Institutes of Health.

## Conflict of Interest

The authors declare that the research was conducted in the absence of any commercial or financial relationships that could be construed as a potential conflict of interest.

## Publisher's Note

All claims expressed in this article are solely those of the authors and do not necessarily represent those of their affiliated organizations, or those of the publisher, the editors and the reviewers. Any product that may be evaluated in this article, or claim that may be made by its manufacturer, is not guaranteed or endorsed by the publisher.
